# Using Bayesian Population Viability Analysis to Define Relevant Conservation Objectives

**DOI:** 10.1371/journal.pone.0144786

**Published:** 2015-12-10

**Authors:** Adam W. Green, Larissa L. Bailey

**Affiliations:** Department of Fish, Wildlife, and Conservation Biology, Colorado State University, Fort Collins, Colorado, United States of America; Southwest University, CHINA

## Abstract

Adaptive management provides a useful framework for managing natural resources in the face of uncertainty. An important component of adaptive management is identifying clear, measurable conservation objectives that reflect the desired outcomes of stakeholders. A common objective is to have a sustainable population, or metapopulation, but it can be difficult to quantify a threshold above which such a population is likely to persist. We performed a Bayesian metapopulation viability analysis (BMPVA) using a dynamic occupancy model to quantify the characteristics of two wood frog (*Lithobates sylvatica*) metapopulations resulting in sustainable populations, and we demonstrate how the results could be used to define meaningful objectives that serve as the basis of adaptive management. We explored scenarios involving metapopulations with different numbers of patches (pools) using estimates of breeding occurrence and successful metamorphosis from two study areas to estimate the probability of quasi-extinction and calculate the proportion of vernal pools producing metamorphs. Our results suggest that ≥50 pools are required to ensure long-term persistence with approximately 16% of pools producing metamorphs in stable metapopulations. We demonstrate one way to incorporate the BMPVA results into a utility function that balances the trade-offs between ecological and financial objectives, which can be used in an adaptive management framework to make optimal, transparent decisions. Our approach provides a framework for using a standard method (i.e., PVA) and available information to inform a formal decision process to determine optimal and timely management policies.

## Introduction

Conserving natural resources requires managers and policy makers to make decisions regarding complex ecological systems. Structured decision making (SDM) and adaptive resource management (ARM) provide a framework for making decisions, while incorporating various sources of uncertainty, and aim to reduce uncertainty through the implementation of a well-designed monitoring program [1–4]. The foundation of these decision analysis frameworks is the articulation of well-defined management objectives, but formulating objectives that best capture the dynamics and response of the ecosystem to conservation actions is not trivial.

Objectives are clear, quantitative metrics that reflect the desired outcomes of stakeholders and are used to guide decisions and measure success [4]. They are used to evaluate the response of the system to conservation actions and are the focus of monitoring programs that are critical to this evaluation [4–6]. A common objective for many management and conservation programs is to maintain a sustainable or viable population [7–10]. This objective is the main focus for federal governments across the globe tasked with protecting species from extinction, including the U.S., with the Endangered Species Act [11] and the Marine Mammal Protection Act [12], Australia [13], Canada with the Species at Risk Act [14], and the European Union’s Habitats Directive. Determining what constitutes a “sustainable” population is often a difficult task that varies from one species to another or even among populations [15]. Still, such definitions are necessary if an ARM framework is employed to differentiate among possible conservation measures.

Population, or metapopulation, viability analysis (PVA) is a common tool used to evaluate sustainability by calculating population persistence in response to changes in demographic parameters. Such analyses can also determine the minimum viable population size necessary for population persistence. The development of PVA corresponded with legislative mandates to sustain viable populations of terrestrial vertebrates [16], and PVA metrics are often used to evaluate the impacts of potential conservation or management actions [17–23]. Martin et al. [24] argue that information about an ecological system is most useful to management and conservation when it is incorporated into models used to derive management decisions, and an ARM framework that incorporates the metrics associated with PVA provides a useful tool for informing conservation decisions [1,2,21,25–26].

Viability assessments of populations or metapopulations have been criticized for making predictions that are imprecise and difficult to test. These criticisms are often attributed to poor data, imprecise parameter estimates and inability to validate models, the lack of separation of sampling and process variance in demographic parameters, and the potential effects of alternative model structures [16,21,23,27–31]. Bayesian population viability analysis (BPVA) addresses some of these shortcomings by using posterior probability distributions for parameters of interest in population projections [32–33]. Bayesian PVA models can be constructed so that posterior distributions represent the process variation in the parameters, rather than the full (undifferentiated) sampling distribution encompassed by most maximum-likelihood approaches, and account for covariation between parameters, allowing one to fully incorporate multiple sources of uncertainty in conservation decisions [34].

Bakker and Doak [35] also developed an approach to address a subset of the criticisms mentioned above; however, their approach only considers one management action at a time and does not incorporate trade-offs between ecological and non-ecological (e.g., cost) objectives. Our approach utilizes a Bayesian metapopulation viability analysis (BMPVA) to help stakeholders determine measurable objectives that can be weighted with other competing objectives (e.g., cost), to reflect the values and risk tolerances of stakeholders. By integrating the BMPVA within an ARM framework, the criticisms of validating models and reducing uncertainty among alternative model structures are naturally, and necessarily, addressed through regular monitoring of the system and assessment of competing models.

Here, we use BMPVA to determine the number of occupied patches (breeding pools) needed to ensure long-term persistence for two amphibian metapopulations. Amphibian populations around the world have declined in recent decades [36–39], and climate change may exacerbate these declines. Many amphibian species use ephemeral pools for breeding, and these species may be disproportionately affected by climate change. Temperatures are expected to increase and precipitation become more variable [40], which may result in shorter hydroperiods for breeding habitats, potentially increasing reproductive failure as habitats dry earlier. Active conservation measures may be needed to mitigate these effects, but it is uncertain how actions may influence long-term persistence. Accordingly, we developed a BMPVA to predict long-term persistence of each metapopulation to determine whether conservation efforts are warranted and, if so, how the metapopulation will respond to potential efforts.

Pond-breeding amphibians are often viewed as a metapopulation because of their strong site fidelity among breeding adults, low dispersal rates, and spatially disjunct breeding sites that vary in quality over space and time [41–43]. Population size at these breeding locations may vary widely over time with frequent complete reproductive failure [44–48]. For these reasons, occupancy surveys are often used to monitor amphibian metapopulations and the factors influencing their dynamics [39, 49–52]. We used breeding and successful metamorphosis occupancy probabilities estimated from existing data to parameterize a BMPVA for each study area. We account for temporal and spatial variation in demographic parameters by using multiple years of data at all or a large sample of breeding habitats and incorporating these data into a model that includes what we believe are the major drivers of the system. We then demonstrate one way to use the BMPVA results to formalize management objectives within a decision framework for the conservation of these species.

## Methods

### Ethics Statement

Sampling occurred with permission from Patuxent Research Refuge and Rock Creek National Park under approval from the U.S. Geological Survey Patuxent Wildlife Research Center’s Animal Care and Use Committee. Occupancy surveys of wood frog egg masses were visual, and the occupancy status of late-stage tadpoles was determined using dip net surveys. All captured individuals were immediately returned to the pool of capture. All sampling procedures were reviewed and approved as part of obtaining the field permits.

### Species’ Biology and Data Collection

Wood frogs (*Lithobates sylvatica*) are widely distributed and range from the southern Appalachians to Alaska [53]. They rely on small, seasonal wetlands (vernal pools) for breeding [54–56], and reside in surrounding upland habitat during the non-breeding season [45,57–58]. In our study areas (description below), adult wood frogs migrate to breeding pools in late February or early March, and metamorphosis occurs in late May or early June. Adult females usually breed for the first time as two-year-olds, and the average life span for this species is thought to be three to five years [44].

Our study areas consisted of two federal land holdings in or near Washington, D.C., USA. Patuxent Research Refuge (PRR) covers 5,280 ha and is located in central Maryland, USA; approximately 75% of the refuge is deciduous forest. Rock Creek Park (ROCR) is a 710-ha National Park within Washington, D.C., USA, consisting of approximately 85% mixed mesophytic forest. A random subset of 53 vernal pools was chosen from approximately 2,200 pools at PRR and all nine of the pools at ROCR were included in our study (see [59] for site selection details). Occupancy surveys were conducted at these pools shortly after wood frog egg masses were laid (i.e., late February–early March) to assess the breeding status each year. Pools were then revisited in late May or early June to determine hydroperiod length and to detect late-stage tadpoles in those pools where eggs were detected. Hydroperiod length was determined by the average time of year when the pond usually dried: short-hydroperiod pools rarely retained water through early June, medium hydroperiod pools retained water through early June in wet years, and long-hydroperiod pools retained water through early June in most years. We assume the presence of late-stage tadpoles (Gosner stage ≥35) represented successful metamorphosis. Sampling occurred from 2006–2010 at PRR and 2008–2011 at ROCR. Details of the occupancy surveys can be found in Mattfeldt et al. [60] and Green et al. [48]. We considered the aggregation of vernal pools at each study area as isolated populations due to the expansive urban areas outside of these protected areas and, therefore, did not expect any immigration of wood frogs into the study areas.

### Model Structure and Parameterization

In previous work, we developed a Bayesian hierarchical model to estimate probabilities of breeding and successful metamorphosis, while accounting for imperfect detection (see [48]). We fit this model to the wood frog detection data from PRR and ROCR to obtain posterior predictive distributions for each parameter that we used in our BMPVA. Key parameters in this model included: $\psi_{it}^{1}$, the probability that pool *i* supports wood frog breeding in year t, and conditional on breeding, $\psi_{it}^{2}$ is the probability that the pool supports successful metamorphosis in the same year. Our analysis revealed that both parameters were influenced by pool-specific characteristics, such as hydroperiod length and spatial arrangement. Pool hydroperiod was an important predictor of breeding ($\psi_{it}^{1}$) and successful metamorphosis probabilities ($\psi_{it}^{2}$) at both study areas. In addition, we distinguished between clustered pools, where multiple pools were within 20 m of one another, and pools that are isolated (>300 m from the nearest pool); we assumed that all pools acted as distinct breeding populations and were connected through dispersal of juveniles. The effect of spatial arrangement could not be assessed in the ROCR data due to small sample size, but analysis of the PRR data suggested that the probability of breeding ($\psi_{it}^{1}$) was surprisingly higher for isolated pools, though the effect wasn't strictly significant [48]. Though contrary to traditional metapopulation theory, this result has been found in other amphibians systems [61–63], possibly due to the acoustic nature of breeding aggregations. Isolated pools likely draw in more individuals because there are fewer nearby breeding sites, potentially increasing breeding occupancy probability. Accordingly, we included a spatial arrangement effect in our BMPVA for the PRR wood frog systems, but not for the ROCR wood frog system (see Methods: Simulations section).

Previous states, namely breeding state (eggs/no eggs) in year *t* -1, and metamorph state in year *t*-2, were also included as pool specific covariates when projecting breeding probability, $\psi_{it}^{1}$, because we thought the female breeding population at a pool (represented by the presence of egg masses) would primarily consist of returning breeding females (represented by breeding occupancy status in the previous year) and first-time breeding females (represented by metamorph occupancy status two years prior). We modeled temporal variation in occupancy probabilities using precipitation values obtained from local weather stations for months most likely to influence breeding habitat (October-February) and successful metamorphosis (March-May).

### Simulations

We used the results from our previous Bayesian analysis, highlighted above, to construct and parameterize a projection matrix model for each metapopulation. Breeding and metamorph occupancy states for year *t* were chosen from the following distributions

$$r_{i,t}∼Bern(\psi_{i,t}^{1})$$ (1)

and

si,t∼{Bern(ψi,t2),ri,t=10,ri,t=0 (2)

where *r*_*i*,*t*_ is the breeding occupancy state for pool *i* in year *t*, $\psi_{i,t}^{1}$ is the breeding occupancy probability for pool *i* in year *t*, *s*_*i*,*t*_ is the metamorph occupancy status for pool *i* in year *t*, and $\psi_{i,t}^{2}$ is the metamorph occupancy probability for pool *i* in year *t*. The metamorph occupancy state is conditional on a pool supporting breeding because the presence of egg masses is necessary for metamorphosis.

We simulated wood frog occupancy dynamics to evaluate metapopulation viability under various circumstances by calculating the probability of quasi-extinction, mean time to extinction, and the proportion of pools producing metamorphs. We defined quasi-extinction as a simulation in which all pools failed to produce metamorphs for two consecutive years. We believe that this accounts for the storage effect [44,64] of surviving adult frogs returning to breed after a year of complete reproductive failure. Female wood frogs are thought to usually breed for the first time in their second year and live three to five years [44,65]; two years of simultaneous reproductive failure at all pools would likely cause substantial declines in the adult population, rendering the metapopulation effectively extinct.

Smaller metapopulations with fewer habitat patches (pools) are subject to a higher probability of extinction due to demographic and environmental stochasticity [66]. Therefore, we considered scenarios using different numbers of pools to investigate the relationships between the number of pools in the system (“pool population size”) and the probability of and time to extinction for the metapopulation. As the pool population size increased, we expected the probability of extinction to approach zero [30,67] and the number of pools producing metamorphs to stabilize. We used the estimated parameter distributions described above from each of the two study areas (Tables 1–3) in combination with six levels of pool population sizes (5, 10, 25, 50, 75, and 100 pools) to create 12 different simulation scenarios.

**Table 1:** Means (SD) of posterior predictive distributions of the probability of vernal pools supporting wood frog breeding (${\hat{\psi }}^{1}$) at Patuxent National Research Refuge, Maryland, USA.

Hydroperiod	Spatial	*r*_*t*−1_	*s*_*t*−2_	2008	2009	2010
Short	Clustered	0	0	0.058 (0.023)	0.033 (0.017)	0.140 (0.053)
		0	1	0.094 (0.095)	0.059 (0.069)	0.192 (0.152)
		1	0	0.125 (0.065)	0.074 (0.044)	0.270 (0.125)
		1	1	0.168 (0.141)	0.108 (0.111)	0.314 (0.194)
	Isolated	0	0	0.148 (0.064)	0.089 (0.049)	0.307 (0.112)
		0	1	0.200 (0.159)	0.132 (0.127)	0.356 (0.206)
		1	0	0.274 (0.121)	0.176 (0.096)	0.482 (0.161)
		1	1	0.319 (0.194)	0.221 (0.170)	0.511 (0.211)
Medium	Clustered	0	0	0.385 (0.107)	0.258 (0.100)	0.611 (0.122)
		0	1	0.426 (0.207)	0.310 (0.200)	0.627 (0.190)
		1	0	0.569 (0.116)	0.422 (0.122)	0.763 (0.108)
		1	1	0.592 (0.177)	0.457 (0.195)	0.774 (0.136)
	Isolated	0	0	0.615 (0.156)	0.477 (0.172)	0.792 (0.123)
		0	1	0.630 (0.206)	0.505 (0.230)	0.795 (0.150)
		1	0	0.766 (0.118)	0.648 (0.150)	0.886 (0.082)
		1	1	0.776 (0.139)	0.664 (0.182)	0.893 (0.083)
Long	Clustered	0	0	0.976 (0.053)	0.960 (0.082)	0.989 (0.028)
		0	1	0.978 (0.055)	0.963 (0.086)	0.990 (0.028)
		1	0	0.989 (0.023)	0.981 (0.039)	0.995 (0.011)
		1	1	0.991 (0.022)	0.984 (0.038)	0.996 (0.010)
	Isolated	0	0	0.989 (0.028)	0.981 (0.045)	0.995 (0.016)
		0	1	0.990 (0.028)	0.982 (0.046)	0.995 (0.013)
		1	0	0.995 (0.011)	0.992 (0.018)	0.997 (0.006)
		1	1	0.996 (0.009)	0.993 (0.015)	0.998 (0.004)

Values were sampled from the entire posterior distribution and used to project the occupancy dynamics of wood frogs in population viability analyses. The breeding occupancy status in year *t*-1 is represented by *r*_*t*−1_ and the metamorph occupancy status in year *t*-2 by *s*_*t*−2_ (0 = absent, 1 = present).

**Table 2:** Means (SD) of posterior predictive distributions of the probability of vernal pools supporting wood frog breeding (${\hat{\psi }}^{1}$) at Rock Creek National Park, Washington, DC, USA.

Hydroperiod	*r*_*t*−1_ ^a^	2009	2010	2011
Short	0	0.061 (0.118)	0.253 (0.246)	0.079 (0.141)
	1	0.098 (0.164)	0.365 (0.336)	0.127 (0.191)
Medium	0	0.478 (0.280)	0.830 (0.191)	0.571 (0.266)
	1	0.555 (0.246)	0.860 (0.182)	0.640 (0.234)
Long	0	0.929 (0.183)	0.991 (0.033)	0.944 (0.166)
	1	0.960 (0.115)	0.993 (0.026)	0.971 (0.090)

Values were sampled from the entire posterior distribution and used to project the occupancy dynamics of wood frogs in population viability analyses. The breeding occupancy status in year *t*-1 is represented by *r*_*t*−1_ (0 = absent, 1 = present).

^a^ Metamorph occupancy state at *t*-2 (*s*_*t*−2_) was not included as a covariate to increase the number of years for which we obtained estimates of occupancy.

**Table 3:** Means (SD) of posterior predictive distributions of the probability of vernal pools supporting wood frog metamorphosis (${\hat{\psi }}^{2}$) at Patuxent National Research Refuge, Maryland, USA and Rock Creek National Park, Washington, DC, USA.

Study area	Hydroperiod	2008	2009	2010	2011
Patuxent	Short	0.119 (0.104)	0.188 (0.145)	0.036 (0.045)	-
	Medium	0.677 (0.112)	0.781 (0.110)	0.335 (0.115)	-
	Long	0.743 (0.106)	0.831 (0.091)	0.415 (0.147)	-
Rock Creek	Short	-	0.109 (0.236)	0.306 (0.352)	0.186 (0.277)
	Medium	-	0.152 (0.278)	0.312 (0.242)	0.173 (0.196)
	Long	-	0.936 (0.142)	0.979 (0.086)	0.981 (0.066)

Values were sampled from the entire posterior distribution and used to project the occupancy dynamics of wood frogs in population viability analyses.

For a given simulation scenario, we projected metapopulation dynamics over 100 years and repeated the projection for 1,000 iterations. For each iteration, the number of pools in each hydroperiod and spatial arrangement category was determined by randomly assigning the categories based on their frequencies in the corresponding study area. For example, to simulate dynamics for the scenario involving 10 pools using the ROCR parameter distributions, the number of pools with short, medium, and long hydroperiods were chosen with probabilities 0.556 (5/9), 0.333 (3/9), and 0.111 (1/9), respectively. For simulation scenarios involving PRR parameter distributions, pools were randomly assigned to categories that represented a combination of hydroperiod length and spatial arrangement with associated probabilities in parentheses: short, clustered (0.444); short, isolated (0.185); medium, clustered (0.222); medium, isolated (0.037); long, clustered (0.074); and long, isolated (0.037).

We included environmental variation in each simulation scenario by randomly assigning one of the years for which we obtained estimates (i.e., 2008, 2009, 2010 for PRR and 2009, 2010, 2011 for ROCR) to each year within each iteration. To account for parameter uncertainty, one value for each of breeding and successful metamorphosis probabilities associated with pool *i* was chosen from the posterior predictive distribution associated with the randomly selected year. For example, if we were conducting an iteration using the parameters estimated from PRR and the first year of the iteration was randomly chosen to be “2009”, all parameter values would be randomly selected from their respective posterior distributions associated with PRR in 2009 (Table 1) to project occupancy states in year 2 within the iteration.

At the end of each iteration, we determined whether the metapopulation went quasi-extinct during the 100 year time horizon, and if so, the year of extinction was recorded. In all cases, we recorded the number of pools producing metamorphs in each year. To summarize all 1,000 iterations for each simulation scenario, we calculated three metrics: (1) the probability of extinction as the proportion of iterations in which the metapopulation went quasi-extinct, (2) the mean year of extinction for iterations in which the metapopulations went extinct, and (3) the average proportion of pools producing metamorphs (hereafter, metamorph occupancy rate, ${\tilde{\psi }}^{2}$) from all iterations in each year of the simulation. For simulation scenarios with a probability of quasi-extinction ≤0.05, we calculated the mean proportion of pools producing metamorphs from years 30–100 to allow the occupancy rate to stabilize and eliminate any variation due to transitions from the starting state to the stable state.

### Incorporating BMPVA results into a decision framework

Incorporating ecological information into a decision analysis allows for the objective evaluation of conservation measures in terms of conservation goals. This process involves defining a utility function that represents a trade-off between all management objectives [24]. Managers of our study areas are concerned that regional climate change predictions of increased temperatures and variable rainfall will lead to reductions of amphibian habitats. They have considered constructing new pools (ROCR) or modifying existing unproductive pools (PRR) to increase the number of pools producing metamorphs to mitigate these anticipated climate-related processes. However, these actions incur cost.

In this case, the biological and financial objectives are competing; managing vernal pools increases the probability of metapopulation persistence and the number of pools producing metamorphs but incurs financial costs. We developed a utility function that captures the trade-offs between the biological objectives quantified by our BMPVA and the costs associated with management actions to provide managers with an objective evaluation of the expected outcomes for each management action.

Because we only simulated metapopulation dynamics for systems with 5, 10, 25, 50, 75, and 100 pools, we interpolated the probability of persistence for other metapopulation sizes. We treated each iteration of the BMPVA as a data point, where *y*_*i*_ = 0 if the metapopulation went extinct and *y*_*i*_ = 1 if it remained extant over the time horizon. We then regressed these data against the metapopulation size using logistic regression in the glm function in Program R [68]. We used the estimated coefficients to predict the probability of persistence for metapopulations consisting of different numbers of pools.

Here, we considered four management actions: 1) do nothing, 2) create 1 pool, 3) create 2 pools, and 4) create 3 pools per year. The maximum value of 3 managed pools was chosen through informal consultations with managers of our two study areas; it reflects the upper limit of annual effort that would be given to this habitat manipulation. Other managers may choose any maximum value to reflect their logistical and habitat constraints. Given our chosen maximum, the utility function represents the gains in metapopulation persistence per pool managed (i.e., costs). We calculated the marginal gains [69] to determine the utility value for each potential management action, given the number of pools in the metapopulation. The marginal gains, *G*_*x*_, for *x* number of pools managed were calculated as:

$$G_{x}=\frac{\phi_{x}-\phi_{0}}{x},$$

where *ϕ*_*x*_ − *ϕ*_0_ is the change in the probability of persistence if *x* pools are created versus doing nothing. Management actions that result in expected ${\tilde{\psi }}^{2}$ greater than the threshold determined by our BMPVA receive a utility value (*U*_*t*_) equal to the marginal gains (i.e., *U*_*t*_ = *G*_*x*_). For actions resulting in expected ${\tilde{\psi }}^{2}$ dropping below the threshold level, there is no value associated with that management action (i.e., *U*_*t*_ = 0).

## Results

### Simulations

**Fig 1:**
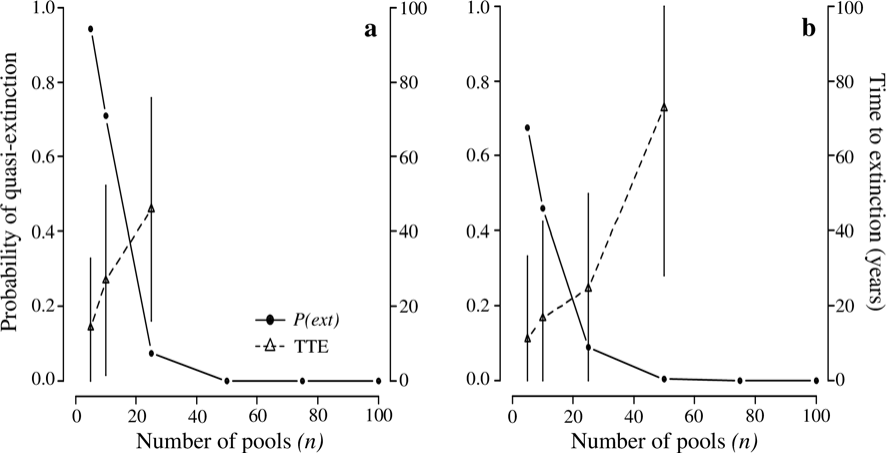
The probability of quasi-extinction (*(P(ext)*) and the mean time to extinction (TTE) for wood frog metapopulations. Simulation scenarios consisted of combining estimated parameter distributions from (a) Patuxent National Research Refuge, Maryland, USA and (b) Rock Creek Park, Washington, DC, USA with different numbers of vernal pools (*n*): 5, 10, 25, 50, 75, and 100 pools. One thousand iterations were run for each scenario. Quasi-extinction occurred when no pools produced metamorphic wood frogs for two consecutive years. Quasi-extinction was not observed in simulations involving >50 pools. Error bars for TTE represent 1 standard deviation.

Our simulations yielded similar results for both the PRR and ROCR wood frog metapopulations. This is likely because, on average, occupancy estimates were similar between the two study areas, which was not initially obvious due to the year-to-year variation in estimates. The probability of quasi-extinction decreased rapidly as the number of pools in the system increased, while the mean time to extinction (TTE) increased with the number of pools in the system (Fig 1). The probability of quasi-extinction fell below 0.05 when there were at least 50 pools in the system using estimates from either PRR or ROCR. The mean TTE for iterations in which the metapopulation went quasi-extinct increased with the number of pools in the system: for PRR scenarios, TTE increased from 14 years for 5 pools to 46 years for 25 pools (i.e., the smallest number of pools resulting in the probability of quasi-extinction >0.05) and from 11 years with only 5 pools to 73 years with 50 pools for ROCR scenarios.

**Fig 2:**
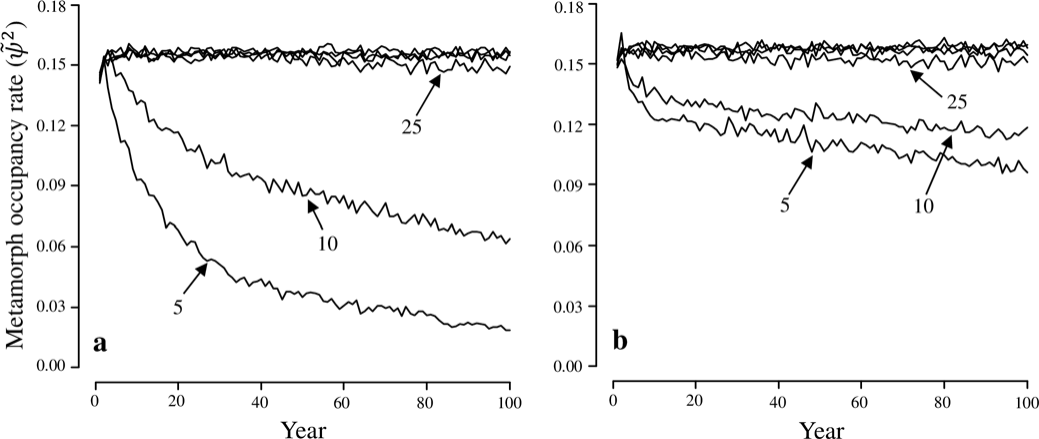
The mean proportion of vernal pools (from 1,000 iterations) supporting wood frog metamorphosis (metamorph occupancy rate, ${\tilde{\psi }}^{2}$) in simulations using occupancy estimates from (a) Patuxent National Research Refuge, Maryland, USA, (b) and Rock Creek Park, Washington, DC, USA, over a 100-year time horizon for scenarios with metapopulation sizes (*n*) of 5, 10, 25, 50, 75, and 100 pools. Labels represent the number of pools in the metapopulation, and overlapping lines represent scenarios for 50, 75, and 100 pools.

The metamorph occupancy rate declined rapidly for scenarios involving a small number of pools, and the magnitude of the decline was slightly different among study areas (Fig 2). The rate of decline was more rapid for PRR scenarios with 5 and 10 pools compared to ROCR scenarios with the same number of pools. However, for systems containing enough pools to ensure long-term persistence (i.e., ≥50 pools, where probability of quasi-extinction ≤0.05), the metamorph occupancy rate stabilized at almost the same value. Using estimates from PRR, the mean metamorph occupancy rate for years 30–100 was 0.156 (SD: 0.002), and similarly, the mean metamorph occupancy rate using estimates from ROCR was 0.158 (SD: 0.002). This suggests that approximately 16% of pools should produce metamorphs to ensure long-term persistence in systems with adequate number of pools. In our wood frog systems, we determined a minimum of 50 pools is necessary to ensure a 0.95 probability of population persistence over 100 years, and approximately 16% of the pools should support wood frog metamorphosis, given the occupancy estimates from either PRR or ROCR.

### Incorporating BMPVA results into a decision framework

**Fig 3:**
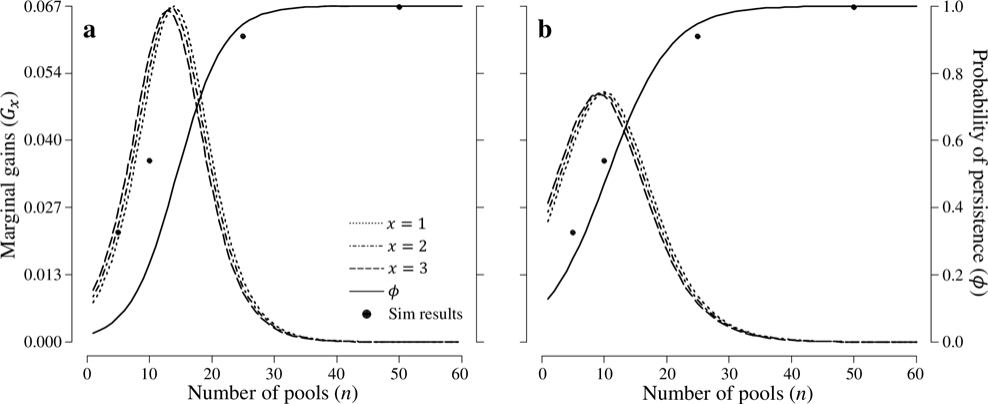
Probability of persistence (*ϕ*) of wood frog metapopulations at (a) Patuxent National Research Refuge, Maryland, USA and (b) Rock Creek Park, Washington, DC, USA, given the number of pools in the metapopulation (n), and marginal gains (*G*_*x*_) associated with the number of pools managed (*x*). Simulation results are represented by closed circles (see Fig 1). Management actions consisted of doing nothing or creating *x* pools.

The predicted probability of persistence as a function of number of pools (*n*) was estimated using logistic regression: *logit*(*ϕ*) = *β*_0_ + *β*_1_ *n*. The coefficients were estimated as *β*_0_ = -3.867 and -2.108 and *β*_1_ = 0.268 and 0.199 for PRR (pseudo-R^2^ = 0.876) and ROCR (pseudo-R^2^ = 0.748), respectively (Fig 3). This results in utility functions of

Ut={Gx,ifψ˜2≥0.1560,ifψ˜2<0.156

and

Ut={Gx,ifψ˜2≥0.1580,ifψ˜2<0.158

for PRR and ROCR, respectively. There are marginal gains from management when *n* > 26 pools (*ϕ* ≈ 0.95). At both sites, the greatest marginal gain is achieved by creating three pools until the number of pools exceeds the inflection point of the persistence curve (~10–13 pools); at this point the optimal action switches to managing one pool.

## Discussion

The concept of adaptive resource management (ARM) has gained wide support recently in the fields of natural resource management and conservation as a framework to make optimal management decisions. ARM allows one to deal with several sources of uncertainty through modeling approaches and well-designed monitoring programs [70]. When taking this approach, clear, measurable management objectives are required [4]. We used posterior parameter distributions obtained from data specific to our study areas in a BMPVA to incorporate correlated environmental and parameter uncertainty into our simulations. This approach allows us to better represent the ecological processes and associated uncertainties by separating sampling and process variance. BMPVA provides a method for defining and quantifying relevant objectives that may be difficult or impossible to directly measure, such as population persistence or viability, by using available information to simulate long-term population dynamics.

Often, decision makers have little information on historical population sizes or ecosystem dynamics, which may relate to management objectives. This uncertainty may lead to reluctance or doubt in defining appropriate biological objectives to use as a measure of ecosystem health. Viability analysis and, more specifically, BMPVA provide a means to quantify these objectives even when data are sparse by projecting the population through time in response to annual changes in environmental conditions or management actions. Incorporating uncertainty in parameter estimates provides a measure of confidence in the ability of a state-variable (e.g., proportion of pools with successful metamorphosis) to represent a management objective (e.g., metapopulation persistence). Incorporating stochasticity into PVAs is important for making appropriate predictions and management decisions [30,71], especially as environmental conditions may change in the future. The iterative nature of ARM provides a useful framework for addressing potential future changes in ecological processes or parameter values because the components of the decision analysis are revisited on a regular basis. New models can be added to the model set to reflect new factors influencing the system, and parameter estimates can be updated as more information is gathered through a monitoring program.

PVA has been criticized for its inability to predict extinction probabilities and minimum viable population size [16] due to poor information on important parameters and the inability to evaluate competing models [21,23,27–29]. Here, we address these elements by developing a Bayesian projection model specific to our biological systems, capturing what we believe are the major drivers of the system, parameterizing the model with estimates based on several years of data, and accounting for both sampling and process variance. Our data revealed high temporal variation among years, especially for short hydroperiod pools; however, we acknowledge that there may be other factors that influence wood frog occupancy dynamics that are not currently included in our simulations. For example, *Ranavirus* (Family: Iridovirus) has been known to cause localized reproductive failure in many amphibian species [72], and occasional die-offs have been observed at select PRR pools (E.H.C. Grant, U.S. Geological Survey, personal communication). The inclusion of additional years of data would likely better capture the inherent variability in vernal pool systems and its impact on wood frog metapopulations. Despite only including three years of estimates in the BMPVA, we believe that it is still justified to use available local data to help make informed management decisions, updating the estimates over time with additional monitoring data. Other studies have also suggested that phenomena, such as inbreeding depression, genetic drift, and genetic bottlenecks, may increase the probability of extinction, especially for small, isolated populations (e.g., [21,73–75]). In a concurrent study, we found dispersal rates of 8–15% from natal pools [65], and Furman et al. [76] found no evidence of differentiation among wood frog populations over a larger spatial scale and more heterogeneous landscape. Therefore, we do not believe that genetic factors have a substantial role in the metapopulation dynamics at our study sites.

Monitoring may be important to identify changes in the hydrology of pools over time. We did not address the non-stationarity of pool hydrology within our simulations, but as the climate changes, we may expect pools to dry earlier, potentially affecting occupancy probabilities. Rather than attempt to model the complex interactions between topography, evapotranspiration, evaporation, soil permeability, and precipitation (e.g., [77–79]), managers could reassess the hydroperiod of pools at longer time intervals (e.g., 10 years) to update pool hydroperiod status, and even incorporate these habitat transitions in simulations to better reflect amphibian-habitat dynamics.

The two study areas highlighted in this paper are part of a larger effort to better manage vernal pool species in the northeastern U.S. (see [59] for other landowners). Our utility function, while realistic, simply represents a template that includes biological and logistical objectives whose values will vary among each participating federal land owner. For example, using our utility function, managers can identify the point at which the gain in persistence is negligible (e.g., *ϕ* > 0.85, *ϕ* > 0.99), such that the increase in persistence resulting from management does not represent an increase in the utility. Alternative utility functions should be developed to represent appropriate stakeholders’ values for each objective in a particular scenario. There is a large body of literature detailing methods for identifying utility functions that capture stakeholders’ values and risk tolerances associated with each objective [80–85]. Examples of these methods include: identifying constraints beyond which an outcome is unacceptable (i.e., metamorph occupancy threshold); pricing out objectives, a technique that identifies how much stakeholders are willing to trade one objective (e.g., cost) to change another objective (e.g., abundance or persistence; [85]); and calculating the certainty equivalent, a guaranteed return stakeholders would accept over a higher, but uncertain, return [82]. These approaches may result in non-linear relationships between objectives and their utility functions depending on stakeholders’ values and their degree of risk aversion.

Likewise, there are numerous methods that combine several single-objective utility functions into an overall utility function [85–87]. Applying constraints to some objectives may reduce the decision problem to a single-objective optimization, which simplifies optimization. For problems where constraints cannot simplify the decision process, each objective can be scaled from 0 to 1 using proportional scoring and then combined into an additive utility function, weighting each objective by stakeholder importance [85–87]. Elicitation techniques, such as swing weighting [85,88] and the Delphi method [89–90], are useful methods for quantifying the relative value (weight) of each objective. Other approaches do not necessarily result in optimal decisions, but identify actions that maximize the minimum gains (maxi-min) or minimize the maximum losses (mini-max; [91–92]), satisfy all stakeholders (satisficing; [93]), or provide a robust decision when there is a large amount of uncertainty (info-gap; [94–96]). In our example of maintaining amphibian populations, each federal land owner must develop their own utility function that represents the values of their stakeholders. These changes may ultimately change management decisions, but the framework used in the decision process is the same.

Stochastic Dynamic Programing (SDP; [97–98]) can be used to determine the decision that optimally balances multiple competing objectives over time. SDP uses the utility function to determine the value of making a particular management decision, given the current state of the system. By projecting system dynamics and management decisions, a decision policy can be found that optimizes all objectives over a long time horizon, while accounting for uncertainty in system dynamics, management efficacy, and model uncertainty.

Using existing data and its associated uncertainties within a BMPVA to quantify difficult to measure or unknown management objectives and incorporate them into utility functions is consistent with the aims of ARM. The most current information is used to make informed decisions and update models as more information is gathered. Future decisions made through ARM are based on the accumulation of information and updated on a regular basis to reduce uncertainty and incorporate changing stakeholder objectives.
