# Correction: Using Bayesian Population Viability Analysis to Define Relevant Conservation Objectives

**DOI:** 10.1371/journal.pone.0147163

**Published:** 2016-01-11

**Authors:** Adam W. Green, Larissa L. Bailey

In the Funding section, the funder “Amphibian Research and Monitoring Initiative” is incorrectly referred to as the “Amphibian and Reptile Monitoring Initiative.” The correct funding information is as follows: This work was funded by the 2008 USGS Amphibian Research and Monitoring Initiative competitive grant program. The funder had no role in study design, data collection and analysis, decision to publish, or preparation of the manuscript.

## References

[1] Green AW, Bailey LL (2015) Using Bayesian Population Viability Analysis to Define Relevant Conservation Objectives. PLoS ONE 10(12): e0144786 doi:10.1371/journal.pone.0144786 26658734 10.1371/journal.pone.0144786 PMC4684342

